# Structure of Spike Count Correlations Reveals Functional Interactions between Neurons in Dorsolateral Prefrontal Cortex Area 8a of Behaving Primates

**DOI:** 10.1371/journal.pone.0061503

**Published:** 2013-04-22

**Authors:** Matthew L. Leavitt, Florian Pieper, Adam Sachs, Ridha Joober, Julio C. Martinez-Trujillo

**Affiliations:** 1 Cognitive Neurophysiology Laboratory, Department of Physiology, McGill University, Montréal, Canada; 2 Institute for Neuro- & Pathophysiology, University Medical Center Hamburg-Eppendorf (UKE), Hamburg, Germany; 3 Division of Neurosurgery, Department of Surgery, The Ottawa Hospital Research Institute, University of Ottawa, Ottawa, Canada; 4 Douglas Institute, Department of Psychiatry, McGill University, Montreal, Canada; University College London, United Kingdom

## Abstract

Neurons within the primate dorsolateral prefrontal cortex (dlPFC) are clustered in microcolumns according to their visuospatial tuning. One issue that remains poorly investigated is how this anatomical arrangement influences functional interactions between neurons during behavior. To investigate this question we implanted 4 mm×4 mm multielectrode arrays in two macaques' dlPFC area 8a and measured spike count correlations (*r_sc_*) between responses of simultaneously recorded neurons when animals maintained stationary gaze. Positive and negative *r_sc_* were significantly higher than predicted by chance across a wide range of inter-neuron distances (from 0.4 to 4 mm). Positive *r_sc_* were stronger between neurons with receptive fields (RFs) separated by ≤90° of angular distance and progressively decreased as a function of inter-neuron physical distance. Negative *r_sc_* were stronger between neurons with RFs separated by >90° and increased as a function of inter-neuron distance. Our results show that short- and long-range functional interactions between dlPFC neurons depend on the physical distance between them and the relationship between their visuospatial tuning preferences. Neurons with similar visuospatial tuning show positive *r_sc_* that decay with inter-neuron distance, suggestive of excitatory interactions within and between adjacent microcolumns. Neurons with dissimilar tuning from spatially segregated microcolumns show negative *r_sc_* that increase with inter-neuron distance, suggestive of inhibitory interactions. This pattern of results shows that functional interactions between prefrontal neurons closely follow the pattern of connectivity reported in anatomical studies. Such interactions may be important for the role of the prefrontal cortex in the allocation of attention to targets in the presence of competing distracters.

## Introduction

Neurons in the primate dorsolateral prefrontal cortex (dlPFC) are arranged in microcolumns spanning approximately 0.7 mm, wherein neurons with similar visuospatial tuning are interconnected with one another [1], [2]. This intrinsic connectivity pattern is thought to allow neurons to functionally interact with one another during behavior. However, measuring such interactions is challenging because one must simultaneously record the responses of multiple dlPFC neurons and compute measurements of functional interactions between them in behaving primates.

One commonly used measurement in primate electrophysiological studies is the Pearson's correlation coefficient (*r*) between the firing rates of two simultaneously recorded neurons [3]. When *r* is computed between neuronal responses over many trials of the same behavioral condition (noise correlations or *r_sc_*), it provides a measurement of functional interactions between two units, which can be due to shared sensory inputs. However, in the absence of sensory inputs, *r_sc_* could provide a measurement of functional connectivity between neurons [4]–[7]. Previous studies have measured *r_sc_* in several cortical areas of the macaque brain and reported a variety of results. In the visual cortex, it seems to be acknowledged that *r_sc_* can hinder visual coding, and that cognitive variables such as attention can decrease *r_sc_* (reviewed in [8]). In prefrontal neurons, however, this picture is less clear. One study in the frontal eye fields (FEF), a prefrontal area involved in the coding of gaze commands, has proposed that *r_sc_* may reflect cooperation and competition between neurons within a network during cognitive tasks such as target selection [9]. The exact topography of such interactions and their dependence on sensory inputs have not been yet well documented.

A previous study used single electrodes to record the responses of dlPFC neurons and reported positively correlated firing during different periods of a delayed-match-to-sample task [10]. The *r_sc_* decreased with the distance between neurons and did not change during the different task periods. However, they did not explore inter-neuron distances larger than 1 mm. Two other studies recorded from the same region have reported similar results [11], [12]. Although these results have revealed that dlPFC neurons functionally interact with one another during behavior, they have also generated important questions. First, over what distances do interactions between dlPFC neurons occur? For example, it is known that microcortical columns in the dlPFC span over a range of ∼0.7 mm but the pattern of collateral connections between dlPFC neurons could extend as far as 7 mm [1]. The results reported by previous studies exploring distances below 1 mm could likely account for interactions between neurons within a microcolumn, but not between microcolumns. According to the pattern of collateral connections, one would anticipate that correlated firing would extend beyond 1 mm. Second, if neurons within the dlPFC interact through both excitatory and inhibitory connections, as suggested by models of the dlPFC circuitry [13], one would anticipate finding both positive and negative correlations. With the exception of one report [14], which also limited their exploration of functional interactions mainly to neurons located 0.2 to 0.3 mm apart, most studies have generally reported positive *r_sc_*. Third, the aforementioned studies used single electrodes and in some cases computed *r_sc_* between units recorded from the same electrode and/or over a few trials. Thus, it is possible that these factors influenced the measured *r_sc_* [8].

In order to investigate these and other related questions, we implanted a microelectrode array in dlPFC area 8a of two macaques and simultaneously recorded the activity of many neurons while the animals kept stationary gaze and waited for the presentation of a visual stimulus. By collecting a large number of trials in the absence of visual inputs into the neurons' RFs, we controlled for common sensory inputs into the units as the source of *r_sc_*. We found that both positive and negative correlations were larger than predicted by chance over a wide range of inter-neuron distances (from 0.4 to 4 mm). Positive correlations were stronger between nearby neurons with visual RFs at similar locations and decreased progressively for neurons farther apart—up to the largest recorded distance of 4 mm. Most importantly, negative correlations were stronger between neurons with dissimilar RF locations and increased as the distance between the units increased.

## Methods

We recorded the responses of neurons in the dlPFC of two behaving adult male *Macaca fascicularis* (“JL” and “F”).

### Ethics Statement

All the experimental procedures were carried out in accordance with Canadian Council for Animal Care guidelines and were pre-approved by the McGill University Animal Care Committee. Animals were pair-housed in enclosures and interactive environmental stimuli were provided for enrichment. During experimental days, water was restricted to a minimum of 35 ml/kg/day, which they could earn through successful performance of the task. Water intake was supplemented to reach this quantity if it was not achieved during the task, and water restriction was lifted during non-experimental days. The animals were also provided fresh fruits and vegetables daily. Body weight, water intake, and mental and physical hygiene were monitored daily. Blood cell count, hematocrit, hemoglobin, and kidney function were tested quarterly. If animals exhibited discomfort or illness, the experiment was stopped and resumed only after successful treatment and recovery. All surgical procedures were performed under general anesthesia. None of the animals were sacrificed for the purpose of this experiment.

### Task

**Figure 1:**
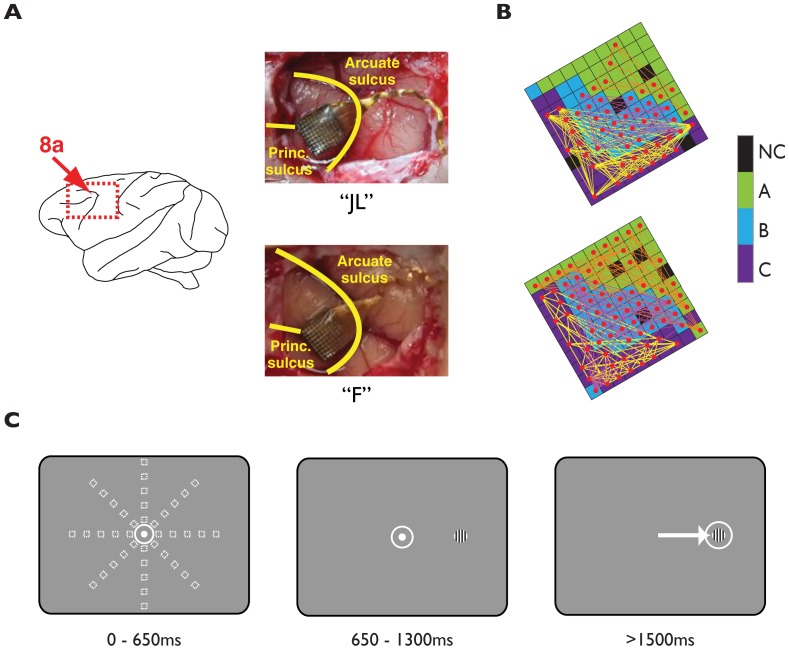
Locations of microelectrode array (MEA) implantations, task design, and correlation pairs on the MEA. **A**) Pictures of the MEA implantation positions. Photographs were taken during the implantation procedure in monkeys ‘F’ and ‘JL’. Principal and arcuate sulci are indicated. **B**) 32-channel recording blocks (‘A’, ‘B’, ‘C’) in each of the two implanted MEAs. Each square represents an electrode. Black squares are not connected (‘NC’). Red dots indicate single units and colored lines represent correlation pairings. **C**) Time-course of events during an example trial. The grey window indicates the screen, the white dot the fixation spot, the squares the positions of the grating, and the white circles the fixation-window.

A custom computer program controlled the stimulus presentation and monitored eye position signals and behavioral responses. The animal initiated a trial by maintaining gaze (fixate) within a 2° window centered on a central fixation spot (0.08 degrees^2^) and pressing a lever; fixation needed to be maintained until the end of a trial. After 650 ms of fixation, a sine-wave grating (2.5 Hz/deg, 1° diameter, vertical orientation) appeared at one randomly selected out of 40 locations (8 directions in 45°-steps, 5 eccentricities in 3°-steps) for 650 ms. After that period, the fixation spot disappeared and the animal had 600 ms to saccade toward the grating (**Figure 1C**). If the saccade landed on the grating, the animal received a juice reward and could initiate the next trial after 1 second. Fixation breaks during the trial or failure to saccade to the target resulted in immediate trial abortion without reward.

### Apparatus and recording procedures

Prior to the experiments, the animals were implanted with 3 head-posts—one placed posterior to the supra-orbital ridge in the midline and the other two on the petrosal bones superior to the external occipital protuberance behind the left and right ears, respectively. The head posts interfaced with a head holder to fix the monkeys' heads to the chair during the recordings. Eye-positions were monitored using an infrared video-based eye-tracker (EyeLink 1000, SR Research, Ontario, Canada) [15].

### Microelectrode array (MEA) implant

We chronically implanted a 10×10 MEA (Blackrock Microsystems LLC, Utah, USA) [16], [17] in each monkey's left dlPFC—anterior to the knee of the arcuate sulcus and caudal to the posterior end of the principal sulcus (area 8a) (**Figure 1A**). Shortly, the surgical operation was carried out under general anesthesia with endotracheal intubation. An incision was made on the scalp with a scalpel and electrocautery. The scalp was retracted and the pericranium excised to limit biological reaction around the implant. A craniotomy was then fashioned using a high power drill (Anspach, FL, USA) over the desired implant location. Wet gelfoam was applied to the epidural edges for hemostasis. The array connector was fixed to the skull with cranial screws and the wires to the arrays were bent appropriately. The wire was secured in place with Silastic (silicon polymer, World Precision Instruments, FL, USA). The dura was opened with a #11 blade and Reynold scissors, and then tacked back with 4-0 vicryl sutures.

After exposure of the brain, the array was placed on the cortical surface and an array gun (Blackrock Microsystems LLC, Utah, USA) was set up in a stereotactic frame. Once properly aligned, the gun was fired, pushing the array ∼1 mm into the cortex. A duroplasty was then done using synthetic dura (Durepair, Medtronic, Inc. Minneapolis, MN, USA), and the bone flap (preserved in saline after the craniotomy) was replaced and secured back using cranial fixation plates and screws (Synthes, Inc. PA, USA). Gaps in the bone were filled with Silastic and the scalp was released from retraction. A small incision was made in the scalp over the connector, to allow the connector to be percutaneous. The scalp was closed in layers with buried vicryl sutures in the galea and staples to the skin. The animal fully recovered from the surgery within one week.

### Recordings and spike detection

Data were recorded using a Cerebus Neuronal Signal Processor (Blackrock Microsystems LLC, Utah, USA) via a Cereport adapter. After 1× amplification in the headstage (ICS-96), the neuronal signal was band-pass filtered (0.3 Hz/1-pole, 7.5 kHz/3-pole, analog) and digitized (16 bit, 1 µV per bit) at a sample rate of 30 kHz. Spike waveforms were detected by thresholding (manually adjusted to ∼−4 to −4.5× noise amplitude) the digitally high-pass filtered (250 Hz/4-pole or 750 Hz/4-pole) raw data.

The extracted spikes (48 samples at 30 kHz, ∼1.6 ms) were re-sorted in OfflineSorter (Plexon, USA) using a T-Distribution E-M Algorithm in 3-dimensional feature space (Plexon Inc, TX). Only re-sorted spikes of single-neurons distinguishable from the multiunit cluster were included in the analysis. The electrodes on each MEA were separated by at least 0.4 mm and were organized into three blocks of 32 electrodes (A, B, C). Data were collected from one block during each recording session.

**Figure 2:**
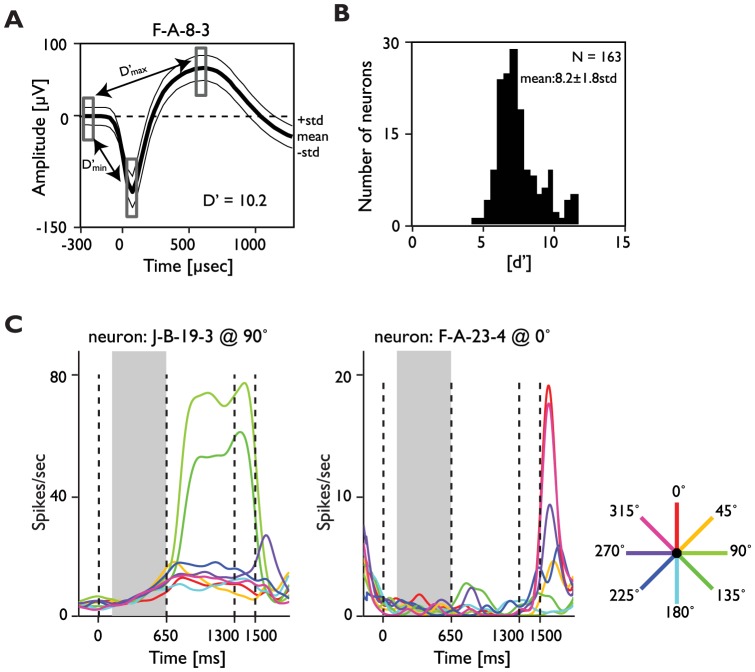
Example neuron, population signal-to-noise and selectivity. **A**) Example average waveform from a recorded neuron (solid line). The abscissa represents time from when the threshold was crossed in microseconds (µs), the ordinate signal amplitude in µV, and the thin lines the standard deviation across waveforms. Rectangles represent the time windows used to measure the standard deviations and to compute d′ (see Methods). **B**) Histogram of d′ distribution across all single-units. **C**) Examples of units' response (spike density function; *σ* = 50 ms) across the different time periods of the task and with different stimulus angular positions (color scale). The gray background indicates the analyzed trial period.

In order to obtain an estimate of the signal-to-noise ratio (separation of the spikes from the background noise) in our recordings, we computed a *d′* index for the distribution of spikes corresponding to each neuron and the associated noise (**Equation 1**).
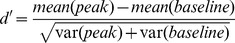
(1)The peak is the minimum/maximum voltage of a given spike distribution, and the baseline is the voltage at the spike onset (noise)—before the first deflection of the waveform (**Figure 2A**). Two *d′* measurements were computed for the first (negative) and second (positive) peaks of the spike waveform. The two were added to obtain a global *d′* measurement of the distance between the peaks and baseline in standard deviation units of the latter (**Figure 2B**).

### Estimation of inter-electrode distance

For electrodes located in the same column or row the interelectrode distance was computed by multiplying the length of a grid segment (0.4 mm) by the number of segments in between the electrodes. For electrodes located in different rows or columns, the Euclidean distance was computed (connecting lines in **Figure 1B**).

### Data analysis

We collected spike data from a total of 163 single neurons (60 in JL, 103 in F) across 6 recording sessions (3 in JL, 3 in F)—one session in each of the MEA's 3 electrode blocks. Neurons with a firing rate of less than 0.1 spikes/second during the 500 ms analyzed period were excluded from the analysis (n = 39), yielding a total of 124 single neurons (34 in JL, 90 in F). We then combined data in the 500 ms period preceding stimulus onset from all correct trials. Next, we grouped units into simultaneously recorded pairs (n = 2003) and computed Pearson's correlation coefficients (*r_sc_*) between the z-scores of the units' spike counts [18]. In addition, we minimized the risk of falsely inflating the correlation values by excluding correlations between units on the same electrode (n = 51) from analysis [8], [18]. These exclusion criteria yielded 1952 correlation pairs for analysis. Fisher's *r*-to-*z* transformation was applied to the correlation coefficients in order to stabilize the variance for hypothesis testing.

### Visuospatial tuning

In order to determine whether a unit was visuospatially tuned for stimulus location, we first computed the mean firing rate for each target location during the 500 ms following the visual stimulus onset. During this period the animal had no information about the upcoming stimulus position and gaze was stationary. We assume that the behavioral state was identical across all trials and the animal was expecting the target onset. Furthermore, the absence of visual stimulation (except for the fixation point, which was the same across trials) and measurable eye movements during that period rule out common visual input into the neurons as the source of spike count correlations. We collected an average of 1010 trials per session (lower limit: 380; upper limit: 1300). We fitted the response data (i.e., mean firing rates) with a circular Gaussian function (**Equation 2**) and computed the goodness of fit (*r^2^*) for each eccentricity [19].
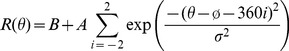
(2)
*R*(*θ*) is firing rate at target angular position *θ*. The parameters *B*, *A*, *φ*, and *σ* represent the baseline, height, preferred target position and tuning width, respectively. We also computed a selectivity index (**Equation 3**) for each neuron.
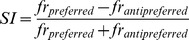
(3)
*fr_preferred_* is the maximum firing rate evoked by a target and *fr_antipreferred_* is the minimum firing rate evoked by a target. Neurons were classified as visuospatially tuned for the target position if the adjusted *r^2^* of the fit was ≥0.75 and the selectivity index was ≥0.5.

We collapsed across eccentricities, as an ANOVA did not show an effect of eccentricity between neurons with similar (≤15°) angular tuning preferences (*p* = 0.740).

#### Control simulations

Variables such as firing rate may affect *r_sc_* [8]. As such, we obtained estimates of *r_sc_* between simulated (independently homogeneously firing) Poisson neurons using the method described in [20] to serve as a control comparison. Briefly, the instantaneous firing rate of a neuron during a 500 ms duration trial was modeled as follows: a) time was subdivided into intervals of *∂t* = 1 ms, b) each interval duration was multiplied by the desired mean firing rate *R* (*∂t*×*R*) to generate 500 identical values (for each Poisson neuron, *R* was estimated according to the firing rate of a matching recorded neuron), c) a sequence of 500 random values (*X(i)*) from a uniform distribution between 0 and 1 was generated, and d) if *X(i)*≤*∂t*×*R* the instantaneous firing rate was set to 1, otherwise it was set to 0. For each neuron, we generated 500 spike trains of 500 ms duration each. This simulation produced a Poisson “mirror” neuron with the same firing frequency as the corresponding recorded neuron. When performing analysis on visuospatially-tuned neurons, the corresponding Poisson neurons were used for comparison. Correlations between pairs of Poisson neurons were also computed to obtain a measurement of correlations due to chance (*r_sc,p_*).

## Results

We analyzed neuronal activity during the 500 ms period preceding the visual stimulus onset (**Figure 1C**). This time period was chosen because: a) it allows measuring of spiking activity during periods of low firing in most of our units during many trials, b) it ensures a common visual input into the neurons RF is not the source of correlated firing, and c) we could compare our results with the ones reported by previous studies in the same area [10]. In animal F spikes were isolated in 70 out of 96 active electrodes (82%), and in animal JL in 52 out of 96 active electrodes (54%) (red dots in **Figure 1B**). The estimated signal-to-noise ratio (*d′*) in the included neurons (n = 163) was 8.2±1.8 SD (see methods and **Figure 2A, B**).

The majority of the recorded neurons showed low spike rates preceding the target onset (**Figure 2C**). Some units showed an increase in firing rate when the visual target appeared at certain locations (left panel), while others did not (right panel). Similar response profiles have been previously described in prefrontal neurons [21].

**Figure 3:**
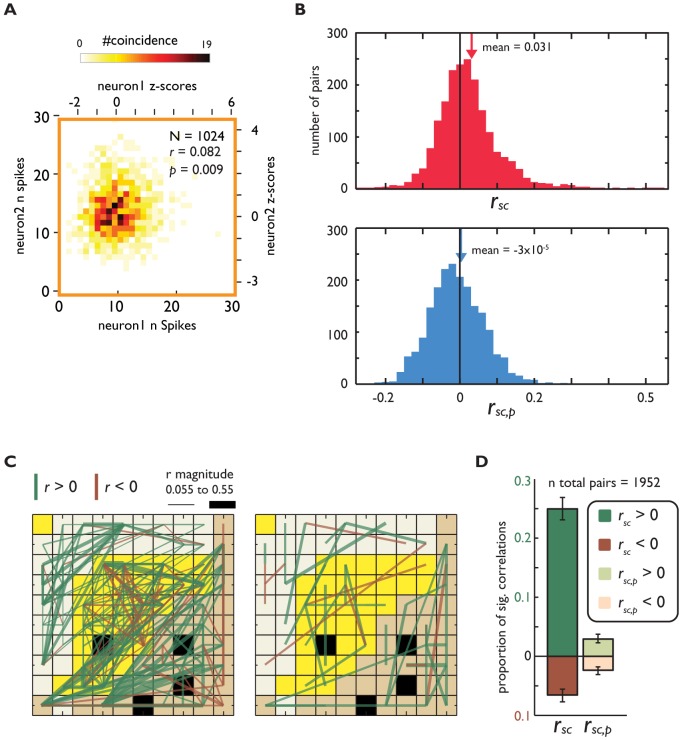
Spike count correlation (*r_sc_*) example and population statistics. **A**) Example correlation between the responses of two neurons. The left x-axis and bottom y-axis represent the absolute number of spikes fired by each neuron in simultaneously recorded trials. The right x-axis and top y-axis show the z-transformed data. The color scale represents coincidences of spike counts. **B**) Histograms displaying the distribution of Fisher-transformed spike count correlations in the recorded data (red) and in the Poisson simulated neurons (blue). The arrows indicate the means of the distributions. **C**) Distribution of correlations and neurons on the MEA from animal ‘F’. Each square represents an electrode, and each line represents a correlation between two neurons recorded on the constituent electrodes. The thickness of a line is proportional to the magnitude of the correlation (from 0.055 to 0.55) and the color represents the sign (brown = negative, green = positive). The panel on the left illustrates significant correlations from the recorded data (*r_sc_*), while the right panel illustrates significant correlations occurring by chance (*r_sc,p_*). **D**) The proportion of significant positive and negative correlations for the data shown in **C**. Observe that the ordinate of the negative correlations is inverted for a better comparison of the correlation magnitudes.

A representative example of *r_sc_* (n = 1024 trials) between the firing rate of two units is illustrated in **Figure 3A**. Both units exhibit spike rates between 5 and 20 spikes per second. The bottom abscissa and left ordinate illustrate the actual firing rates, and the top abscissa and right ordinate show the corresponding z-transformed rates. For these two units, the estimated *r_sc_* was 0.08 and was significantly different from zero (see Methods, *p* = 0.009, *t*-test). We followed the same procedure for each one of the recorded units. The mean of all Fisher-transformed correlations (**Figure 3B**, red histogram) is significantly larger than zero (mean *r_sc,all_* = 0.031, *p*<0.001, *t*-test). The mean chance correlation (blue histogram) was not significantly different from zero (*r_sc,p_* = 3.0×10^−5^, *p* = 0.958, *t*-test; **Figure 3B**, bottom panel) and was significantly smaller than *r_sc,all_* (*p*<0.001, *t*-test, Bonferroni corrected). This shows that *r_sc,all_* in our sample was higher than expected by chance.

In order to better visualize the *r_sc_* between different neurons in the MEA, we plotted the position of each unit as it was recorded on the MEA and joined significantly correlated pairs of neurons with a line. We then grouped the *r_sc_* by sign—into positive and negative. **Figure 3C** illustrates *r_sc,all_* (left) and *r_sc,p_* (right) in three example networks recorded during different sessions (beige, yellow, and light brown backgrounds in the MEA layout). The proportion of significant positive *r_sc_* was significantly larger than that observed by chance (0.250 vs. 0.029; *p*<0.001, chi-square test, Bonferroni corrected). Interestingly, the proportion of significant negative *r_sc_* was also significantly larger than predicted by chance (0.066 vs. 0.020; *p*<0.001, chi-square test, Bonferroni corrected) (**Figure 3D**, bar graph). The proportion of positive *r_sc_* was also significantly larger than the proportion of negative *r_sc_* (*p*<0.001, difference of proportions from the same survey, Bonferroni corrected [22]).

### Influence of distance between neurons and tuning properties on *r_sc_*

**Figure 4:**
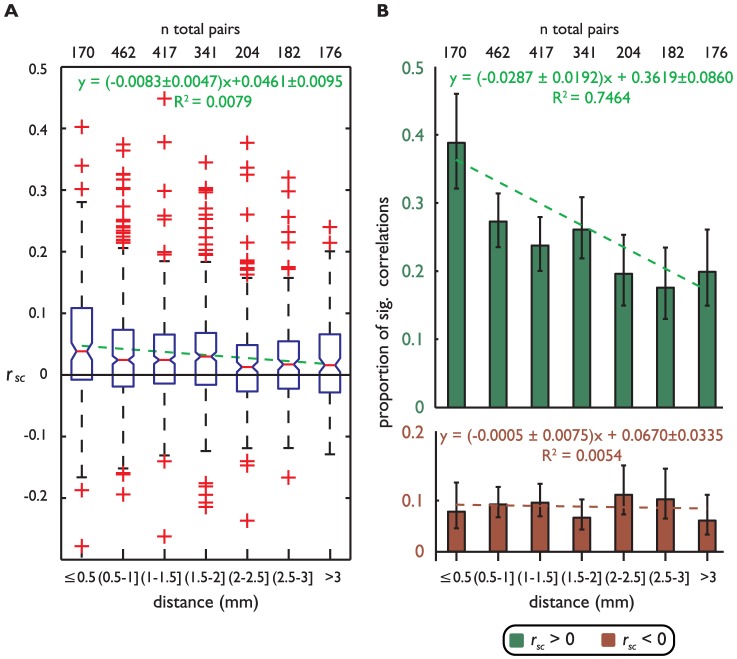
Spike count correlations between all neurons as a function of inter-neuron distance. **A**) Distribution of Fisher-transformed *r_sc_* for all correlations (ordinate) as a function of distance between neurons (abscissa). Each bar indicates the median (red line), the box indicates the center 50%, and the whiskers extend to approximately ±2.7σ. Crosses indicate outliers beyond this interval. The green line and equation represent the best line fit through the data. The 95% confidence intervals for the slope are indicated. The total number of pairs is shown on the top **B**) Proportion of significant positive and negative correlations as a function of distance between units for *r_sc_* (see legend). The green and red lines represent best-fit lines for these proportions. The equations for the two lines and 95% confidence intervals for the slopes are shown. There is a significant negative effect of distance on the proportion of significant positive correlations, and no significant effect of distance on the proportion of significant negative correlations (*p* = 0.019 and *p* = 0.876, respectively, F-test for both). The error bars indicate the 95% confidence intervals for the proportions. The total number of pairs is shown on the top.

Previous studies have found that for prefrontal neurons separated by less than 1 mm, the distance between them (inter-neuron distance) influences *r_sc_* during periods of fixation [10]. Here, we examined whether this result can be generalized to longer distances. Indeed, after discretizing the distances into 0.5 mm bins, we found a significant decrease in mean *r_sc_* with increasing inter-neuron distance (*p*<0.001, F-test, see equation of the line fit to the data in **Figure 4A**). Additionally, we examined whether the proportion of significantly correlated pairs changed as a function of distance (**Figure 4B**). For this analysis, we again divided the significantly correlated units by the sign of the correlation. We observed a significant decrease in the proportion of significant positively correlated pairs as distance increased (*p* = 0.019, F-test, see linear equation and green dashed line in **Figure 4B**, top plot). For negative *r_sc_*, the proportion of significantly correlated pairs did not change as a function of distance between neurons (*p* = 0.876, F-test see linear equation and brown dashed line in **Figure 4B**, bottom plot).

**Figure 5:**
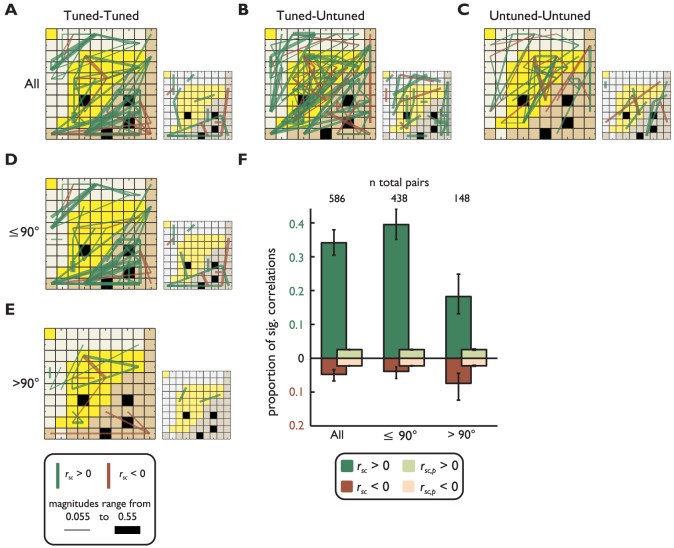
Spike count correlations sorted by neurons' visuospatial tuning preferences. Like **Figure 3**, correlations are visualized on the MEA. Each square represents an electrode, and lines between the electrodes represent correlations between neurons recorded on those electrodes. The thickness of the line is proportional to the magnitude of the correlation (from 0.055 to 0.55) and the color represents the sign (brown = negative, green = positive). The large panels represent *r_sc_* and the small panels *r_sc,p_*. The row displays data from **A**) tuned-tuned, **B**) tuned-untuned, and **C**) untuned-untuned pairs. The column displays data from **A**) all tuned pairs, **D**) tuned pairs with RFs separated by less than or 90°, and **E**) RFs separated by more than 90° (bottom). **F**) The bar graph displays the proportion of significant correlations for all the tuned-tuned pairs (see legend).

Another variable that may affect *r_sc_* is the neurons' tuning for the stimulus position [10]. To examine this issue we divided our sample into neurons tuned for the position at which the stimulus would later appear, and untuned neurons (see **Methods**). We then organized the neuronal pairs into three categories: a) both neurons were tuned, b) one neuron was tuned, and c) neither neuron was tuned. Correlations between tuned pairs were further subdivided by angular difference in preferred stimulus location (RF location): a) neurons with a difference in RF location of either ≤90° (‘similar’ preference group) or b) >90° (‘dissimilar’ preference group). **Figures 5A–E** depict the same example networks from **Figure 3** decomposed using these two criteria (large panels). At least three main observations can be made in **Figure 5**: First, the amount of significant *r_sc_* is larger when at least one neuron of a correlation pair is tuned relative to when both neurons are not visuospatially tuned. Second, the amount of significant *r_sc_* in tuned neurons decreases for neurons with distant RF locations (large panels in first column). Third, there are fewer correlations expected by chance than the quantity observed in the sample (small panels nearby the large ones).

The mean *r_sc_* between tuned units (*r_sc,tuned_*) was significantly larger than in the entire sample (*r_sc,all_* = 0.031, *r_sc,tuned_* = 0.050, *p*<0.001, *t*-test, Bonferroni corrected). This finding was not due to significant differences in firing rate between tuned neurons and the whole population [23] during the analyzed period (*p* = 0.706, *t*-test). Thus, we concentrated on the subset of correlations in which both neurons were tuned (*r_sc,tuned_*). In this group the mean *r_sc,tuned_* was significantly larger than that predicted by chance (*r_sc,tuned_* = 0.050, *r_sc,tuned,p_* = 0.0002, *p*<0.001, *t*-test, Bonferroni corrected).

When separating the tuned pairs based on difference between angular RF location, we found that *r_sc_* in the ‘similarly tuned’ group (*r_sc,similar_* = 0.062) were significantly larger than those in the ‘dissimilarly tuned’ group (*r_sc,dissimilar_* = 0.012, *p*<0.001, *t*-test, Bonferroni corrected). More importantly, both the ‘similar’ and ‘dissimilar’ groups had a significantly larger proportion of significant, positive *r_sc_* than predicted by chance (*p_positive,similar_*<0.001 and *p_positive,dissimilar_*<0.001, chi-square test and Bonferroni corrected for both groups), but only the ‘dissimilar’ group had a significantly larger proportion of significant, negative *r_sc_* than predicted by chance (*p_negative,similar_* = 0.058 and *p_negative,dissimilar_*<0.001, chi-square test and Bonferroni corrected for both groups) (bar graphs in **Figure 5F**). Furthermore, the ‘similar’ group had a larger proportion of significant positive *r_sc_* than the ‘dissimilar’ group (*p*<0.001, chi-square test, Bonferroni corrected). While there was no significant difference between the proportion of significant negative correlations in the ‘similar’ and ‘dissimilar’ groups (*p* = 0.487, chi-square test, Bonferroni corrected), overall the proportion of significant positive *r_sc_* decreased and the proportion of significant negative *r_sc_* increased in the ‘dissimilar’ relative to the ‘similar’ group.

**Figure 6:**
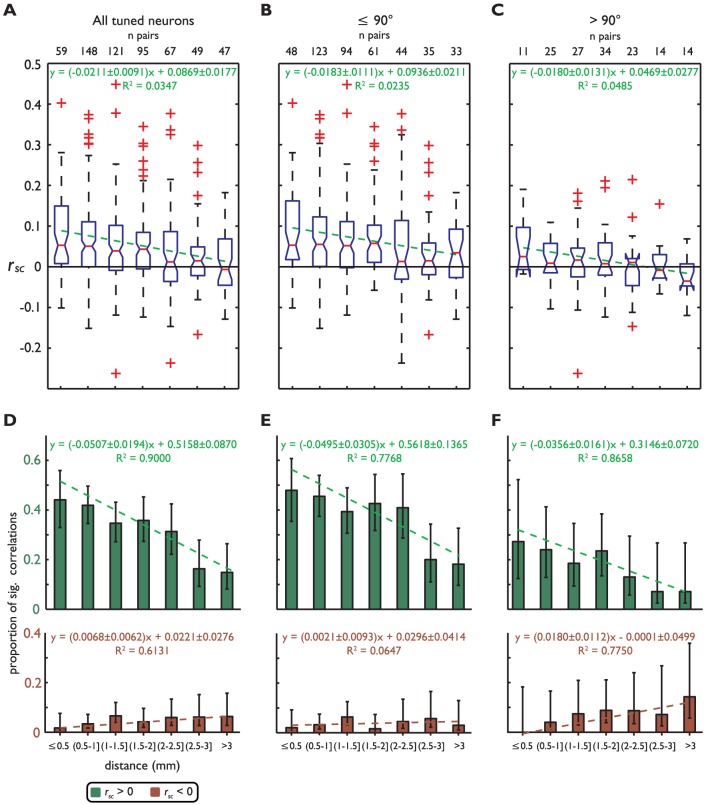
Spike count correlations between visuospatially-tuned neurons as a function of inter-neuron distance. Distributions of *r_sc_* (ordinate) as a function of distance between neurons (abscissa). **A**) Displays all neurons, **B**) neurons with RFs≤90° apart, and **C**) neurons with RFs>90° apart. **D–F**) Proportion of significant positive and negative *r_sc_* and *r_sc,p_* (ordinate, see color legend) as a function of distance between neurons (abscissa), organized by distance between RF as in **A–C**. All symbols are the same as in **Figure 4**.

We examined the relationship between inter-neuron distance and *r_sc_* in tuned pairs. In this group the linear decrease in correlation as a function of distance has a larger y-intercept than that for the whole population (compare **Figure 4A** and **Figure 6A**, *p*<.01, comparison of Bonferroni corrected 95% confidence intervals). When dividing the selective correlations into the ‘similar’ and ‘dissimilar’ RF groups, we did not find a significant difference between the slope or y-intercept of the fitted lines (**Figures 6B and 6C**, *p*>.05, comparison of Bonferroni-corrected 95% confidence intervals). However, the line fitted to the ‘dissimilar’ group indicates negative values of *r_sc_* for the most physically distant pairs, suggesting that the firing of physically distant neurons with dissimilar RF locations is negatively correlated. While the medians of the two largest distance bins were negative, they were not significantly negative, likely due to the small sample sizes (n = 14 for both distances, *p* = .99, *p* = .079 for the second largest and largest distances, respectively, Wilcoxon signed rank test).

We also computed the proportion of significantly correlated pairs as a function of distance. Tuned neurons showed a decrease in the proportion of significant positive *r_sc_* as a function of inter-neuron distance (*p* = 0.001, F-test, see equation in **Figure 6D**, top panel). Interestingly, the proportion of significant negative *r_sc_* increased as the distance between units increased (*p*<0.037, F-test, see equation in **Figure 6D**, bottom panel), a trend not observed in the sample that includes non-selective neurons.

The proportion of significantly correlated pairs was again divided into ‘similar’ and ‘dissimilar’ groups based on the distance between the two neurons' RFs. We found that for the ‘similar’ group the proportion of significant positive correlations decreased as neurons became farther apart (*p* = 0.009, F-test see equation in **Figure 6E**, top panel). However, the proportion of negative correlations showed no significant trend (*p* = 0.580, F-test, see equation in **Figure 6E**, bottom panel). For pairs with RFs located more than 90° apart we observed that the proportion of significant positive correlations decreased as a function of distance (*p* = 0.002 F-test, see equation in **Figure 6F**, top panel). What may be the most surprising result regards the proportion of significant negative correlations, which increases as a function of distance (*p* = 0.009, F-test, see equation in **Figure 6F**, bottom panel). To our knowledge, this is the first report in this cortical region of significant negative correlations that increase in frequency as a function of inter-neuron distance over distances larger than 1 mm.

### Dissociating proximity and tuning similarity

In order to dissociate the effects of inter-neuron distance and tuning similarity on *r_sc_* we applied a multiple linear regression procedure using inter-neuron distance (in mm), RF location difference (in degrees), and the interaction of inter-neuron distance and RF location difference as the predictor variables, and *r_sc_* as the response variable. We found that inter-neuron distance (ß_1_ = (1.87±1.22)×10^−2^) and RF location difference (ß_2_ = (4.97±2.97)×10^−4^) were both significant predictors of *r_sc_* but that the interaction of the two variables was not (ß_3_ = (1.48±16.12)×10^−5^). The y-intercept was significantly greater than zero (ß_0_ = 0.107±0.022) and the overall model fit was *R^2^* = 0.097 (*p*<0.001). This result indicates that the each variable alone is a predictor of *r_sc_*, however their multiplicative interaction is not.

## Discussion

Our study measured spike count correlations between the firing of macaque area 8a neurons using a chronically implanted MEA. We found that in the absence of visual stimulation: a) there were significant positive and negative correlations between neurons that extend over distances ranging from 0.4 to 4 mm; b) such correlations were significantly stronger between visuospatially-selective neurons; c) neurons with RFs ≤90° apart show a significantly larger proportion of positive correlations than units with RFs>90° apart, and such correlations decreased in magnitude as a function of inter-neuron distance; and d) neurons with RFs>90° apart showed a relatively larger proportion of negative correlations than those with RFs≤90°, and such correlations increased in magnitude as a function of inter-neuron distance.

### Previous correlation studies in dlPFC

Although several studies have examined spike count correlations in different brain areas (reviewed in [4] and [8]), only a few studies have measured spike count correlations in dlPFC area 8a of behaving monkeys [10]–[12], [24]. So far, we have not found any study in this brain area that has recorded from multiple neurons using MEAs chronically implanted in the cortex. MEAs have several advantages relative to single electrode recordings. First, they allow precise identification of the targeted brain area because sub-dural implantation is done while visualizing the anatomical landmarks that identify the area (arcuate and principal sulci, [25]). Second, they allow a precise mapping of the recording sites relative to such landmarks that does not change within or between sessions, and thus facilitates the anatomical reconstruction of the explored area (**Figure 1A and B**). Third, they provide stable isolation of single units during a session since the arrays are fixed on the cortex rather than attached to the skull (as during acute single electrode recordings) [17]. This guarantees that small movements of the brain relative to the skull do not produce movement of the electrodes that can change the neurons' isolation or damage the tissue.

Unlike previous studies, we explored correlations between neurons located as far as 3–4 mm apart. For example, [10] and [14] explored inter-neuron distances of up to 1 mm, and [12] distances up to 0.5 mm. We measured correlations between neurons separated by distances from 0.4 to 4 mm. Considering that the width of a prefrontal microcolumn is about 0.7–0.9 mm [1], [26], these studies could not isolate functional interactions between neurons located in adjacent microcolumns but likely limited their findings to neurons located within the same or in nearby microcolumns. Our results agree with the results reported by these authors for distances smaller than 1 mm. Moreover, we found that the proportion of both positive and negative correlations were greater than zero for distances up to 3 mm and higher. Interestingly, negative correlations only became apparent between tuned neurons at distances greater than 1.5–2 mm; hence, these correlations could not be observed in the aforementioned studies.

A novel finding of our study is the pattern of negative correlations shown in **Figure 5** and **Figure 6**. Proportions of negative correlations significantly higher than predicted by chance were mainly found between pairs of neurons with RFs located more than 90° apart (**Figure 5**). Remarkably, such a proportion increased with the distance between neurons (**Figure 6**). A previous study has reported negative correlations between FEF neurons [9] with non-overlapping RFs but did not quantify the trend that correlations increase as a function of interneuron distance. One factor that may have contributed to the novelty of our results is that previous studies used a smaller number of trials, thus reducing the power of statistical tests. For example Constantinidis and Goldman-Rakic [10] used an average of ∼10 trials; Sakurai and Takahashi [12] used a larger number of trials (240 to 270), but they did not explore distances beyond 1 mm. We used an average of 1010 trials and explored a wider range of distances. The increase in the proportion of significant negatively correlated pairs with increasing distance between units cannot be an artifact associated with trial number since this variable was similar for all unit pairs across distances.

Because negative correlations occurred in the absence of visual inputs into the neurons RFs when the animals had no information about the upcoming stimulus location, they likely indicate direct or indirect interactions between neurons rather than a common sensory input to their RFs. These interactions may reflect an anatomical and functional organization of area 8a (i.e., units closer together with similar coding preferences share excitatory connections, and units far apart with dissimilar preferences share inhibitory connections; [2]). It may be possible that the correlations were due to inputs from other non-sensory brain areas reflecting preparation for the task and/or the attentional state of the animal. Although this is a possibility, such inputs must be organized according to the neurons' preferences within area 8a (e.g., RF similarity) and according to the distance between them to produce the pattern of correlations isolated in our data. Thus, we consider it more likely that the isolated pattern reflects the intrinsic functional connectivity between neurons within the area.

One previous study used crosscorrelation techniques to explore functional interactions between neurons located 0.2 to 0.3 mm apart and reported a trough in the crosscorrelation histogram for pairs of neurons with similar tuning [14]. They interpreted this finding as evidence of inhibitory interactions between units that may play a role in functions such as memory maintenance. In our study we found that negative correlations between tuned neurons located closer together were near chance values (see **Figure 6**). However, we did not explore inter-neuron distances as short as 0.2 mm. It is possible that the short-range inhibitory interactions reported by these authors are limited to such short distances. This issue may require further investigation.

More importantly, our results indicate that functional interactions between dlPFC area 8a neurons extend beyond the previously reported distance of 0.9–1 mm [10]. Since the width of a dlPFC micro-column has been estimated as approximately 0.7–0.9 mm [1], [27], our results may reflect functional interactions between different microcolumns, and agree with reports of dlPFC anatomical modules [13], [24], [28], [29]. Moreover, the pattern of positive and negative correlations shown in **Figure 6** suggests that neurons in nearby microcolumns mainly interact through excitatory connections while neurons in clusters far away interact through inhibitory connections. These may underlie the competitive interactions between neuronal representations that are thought to play an important role in target selection and the allocation of attention [30]–[34].

### Comparison with studies of spike count correlation in other brain areas

The majority of studies examining spike count correlations in primates have been conducted in visual areas ([35]–[40]; see [8] for a review). Our results share similarities and differences with the results reported by these studies. For example, the decrease in positive correlations as a function of distance between neurons seems ubiquitous, as well as the changes in correlations as a function of neuronal tuning properties. Our results also agree with those of a previous study in area V1 that reported correlations extending over several millimeters [37]. However, different from our study, they mainly reported positive correlations. This apparent discrepancy with our results may be explained by differences in the intrinsic connectivity pattern of areas 8a and V1. For example, the granular structure and connections with thalamic nuclei differ between visual and prefrontal cortices [41], [42].

In visual areas, correlated firing between neurons with similar coding preferences may hinder stimulus coding [5]. A solution to this problem may be to decrease the correlated firing during behavioral tasks through mechanisms such as attention and learning [43]–[45]. However, studies in the dlPFC have not reported such changes in correlation across behavioral states [10]–[12], [24]. Interestingly, a study in the FEF (located posterior to our recording sites) reported a pattern of negative and positive correlations as a function of RF distance between units and suggested this pattern reflects cooperation and competition between neurons during target selection [9]. Further investigation is needed to clarify whether correlations in the dlPFC change as a function of behavioral states.

Interestingly, it has been proposed that in the visual cortex the structure of the correlations rather than their absolute value determines choice probability during certain tasks [46]. Our results identify a correlation structure in area 8a that seems to reflect the pattern of direct or indirect connectivity between neurons. This basic correlation structure may change during behavior depending on the input signals and computations performed by individual neurons and the entire area network.

### Factors that affect spike count correlations

Several factors may affect the computation of spike count correlations [8]. Amongst them are: response strength, time period for counting spikes, spike sorting, and fluctuation in behavioral state. Differences in response strength between neurons cannot explain the pattern of correlations reported in our study since neuronal firing rates did not differ across distances and RF locations. The time period used to count the spikes also cannot explain our results, because it was identical for all pairs. Spike sorting errors are unlikely the explanation to our results. First, we have provided a measure of signal-to-noise ratio in our sample of recorded neurons. Second, we excluded neurons recorded from the same electrode from the analysis. Finally, one may argue that we may have measured multiunit activity and therefore inflated the correlations. Although this cannot be fully ruled out, we consider it unlikely because we only included units with waveforms that were clearly classified as single neurons by the sorting algorithm. Nevertheless, even if such a factor may have influenced our results, the pattern of positive and negative correlations across distances cannot be explained by spike sorting errors. Fluctuations in the animals' behavioral state also cannot explain our findings since our task was the same across all recording sessions.

In sum, our results demonstrate a pattern of spike count correlations as a function of physical distance between units and distance between their RFs in dlPFC area 8a. They suggest that functional interactions between neurons in this area extend over multiple millimeter distances, likely reflecting the interactions between cortical micro-columns. These may facilitate, under certain behavioral conditions, the competition between neurons holding neural representations of different objects or locations within the area topographic map.
